# Single-Cell Isolation and Gene Analysis: Pitfalls and Possibilities

**DOI:** 10.3390/ijms161125996

**Published:** 2015-11-10

**Authors:** Kjetil Hodne, Finn-Arne Weltzien

**Affiliations:** Department of Basic Sciences and Aquatic Medicine, Norwegian University of Life Sciences—Campus Adamstuen, 0033 Oslo, Norway; kjetil.hodne@nmbu.no

**Keywords:** single-cell, harvesting, RT, amplification qPCR, analysis

## Abstract

During the last two decades single-cell analysis (SCA) has revealed extensive phenotypic differences within homogenous cell populations. These phenotypic differences are reflected in the stochastic nature of gene regulation, which is often masked by qualitatively and quantitatively averaging in whole tissue analyses. The ability to isolate transcripts and investigate how genes are regulated at the single cell level requires highly sensitive and refined methods. This paper reviews different strategies currently used for SCA, including harvesting, reverse transcription, and amplification of the RNA, followed by methods for transcript quantification. The review provides the historical background to SCA, discusses limitations, and current and future possibilities in this exciting field of research.

## 1. Introduction

Genes are regulated at the single cell level, and the stochastic nature of genes turning on and off results in a temporally heterogeneous gene expression, even within homogenous cell populations [1,2,3,4,5,6,7,8,9]. This unique feature is often concealed behind average quantification in whole tissues. Based on earlier discoveries of gene expression dynamics, along with recent improvements in robust and sensitive methods, interest in single-cell omics is growing rapidly. In 2013 single-cell sequencing was awarded “method of the year” by Nature Methods [10] demonstrating groundbreaking discoveries and exciting potential in cell biology [11,12,13,14,15]. Today, novel technologies, like lab-on-a-chip, have facilitated large-scale screenings of transcripts within single-cells. This review opens with a historical perspective focusing on nucleic acid amplification and single-cell gene analysis. We then move on to discuss pros and cons regarding different strategies for harvesting and isolation of nucleic acids, and quantification of gene expression, and finally provide some thoughts on future possibilities within the field of single-cell gene expression.

## 2. Historical Background—Nucleic Acid Amplification

The idea of isolating and analyzing small levels of nucleic acids goes back almost five decades. During the work of unraveling the genetic code, working as a researcher in Har Gobind Khorana’s laboratory, Kjell Kleppe described for the first time a method for primer-defined enzymatic replication of short DNA fragments. However, at that time little focus was put into Kleppe and Khorana’s vision in which a system could target and amplify a specific DNA sequence defined by complementary primers [16]. In fact, it took another decade until Kary Mullis reintroduced the concept of primer-dependent DNA amplification, which we now know as PCR [17]. Through several studies, and subsequent publications, Mullis and Saiki reintroduced and refined Kleppe’s ideas and described the basic principles of exponential DNA amplification employing two complementary primers for each DNA strand [18,19]. The initial PCR protocol consisted of 20–27 cycles with 2 min at 95 °C to separate the two DNA strands followed by 2 min at 37 °C, allowing the primers to anneal and the polymerase to synthesize the complementary strand. However, because of the thermolability of the polymerase (Klenow fragment of *Escherichia coli* DNA polymerase (I)), it was inactivated during the 95 °C step. As a result, the procedure required new polymerase between each cycle. This limitation was overcome a few years later when Saiki *et al.* [20] utilized a thermostabile DNA polymerase [21] isolated from *Thermus aquaticus* [22]. With this refinement, scientists could conduct the DNA amplification reaction at high temperatures without adding new enzyme between each round of the PCR cycle. The higher amplification temperature also permitted more precise targeting of the DNA and reduced the incidence of primer dimers. Combined with the *in vitro* development of reverse transcription (RT) of mRNA into complementary DNA (cDNA) [23,24,25,26], detailed investigations of target transcripts became feasible. In order to visualize the PCR product(s), the samples were separated using gel-electrophoresis [27,28,29].

The sensitivity of PCR was clearly demonstrated by Li *et al.* [30] who, in 1988, analyzed genomic DNA of single sperm cells collected through a glass capillary. Two years later, Brady *et al.* [31] were able to analyze gene transcripts from single macrophages. This ability, to amplify and analyze transcripts from single cells, was taken one step further when Eberwine *et al.* [32,33] and Lambolez *et al.* [34] combined patch-clamp recordings with single-cell RT-PCR. Eberwine *et al.* utilized acutely dissociated neuronal cells obtained from the hippocampus of neonatal rats. The patch pipette served two purposes: to deliver oligo(dT) (with T7 recognition), deoxynucleoside triphosphates (dNTPs) and RT enzyme (Avian myeloblastosis virus), and to insulate the electrode solution needed to perform the electrophysiological recordings. Following the electrophysiological recordings, negative pressure was applied through the patch pipette and the cytosol was carefully collected with the pipette for nucleic acid amplification. In these experiments, several rounds of pre-amplification using T7 RNA polymerase in isothermal conditions were performed to increase the transcript concentration prior to the PCR. This approach allowed Eberwine *et al.* [33] to qualitatively detect transcripts of specific Ca^2+^ channels, γ-aminobutyric acid (GABA) receptors, K^+^ channels, and Na^+^ channels, as well as G-protein subunits and transcription factors c-jun and c-fos. The same group also conducted semi-quantitative measurements of transcript levels by measuring the relative intensity of the ethidium bromide (EtBr)-stained PCR products at the end of the PCR. However, as will be explained in the following sections, this method is unreliable and the quantitative results should be interpreted with caution. Lambolez *et al.* [34] used a slightly different approach to characterize several forms of AMPA receptors and their splice variants. Instead of pre-amplification of RNA, two rounds of PCR were conducted. Following the first round of PCR to amplify large fragments of the cDNA template, internal or nested primers were used to amplify a smaller fragment from the first PCR product. Similar to pre-amplification using T7 RNA polymerase, the PCR pre-amplification strategy also increases the amount of product needed to detect low abundance transcripts. 

Although the technical difficulty of investigating low-level transcripts was now resolved, the challenge of quantitatively measuring transcript levels remained. Traditionally, gene quantification was performed at the so-called plateau phase of the PCR at the end of a PCR assay (semi-quantification). However, as discovered by Higuchi and co-workers [35,36] this plateau phase differs among replicated samples and was first discovered when Higuchi and co-workers started experimenting with the possibility of monitoring the PCR continuously, or in real-time during each amplification cycle [35,36]. By adding EtBr to the PCR reaction and using a charge-coupled device (CCD) camera, every PCR cycle could be monitored as a function of increasing fluorescence. It was clearly shown that after the initial exponential phase, the PCR enters a linear phase followed by a plateau phase [36,37,38]. This plateau phase results from inhibition of the PCR [37,38] and sample-to-sample variation, causing imprecise quantitative calculations [39]. When monitoring the PCR in real-time (*i.e.*, at each cycle), however, it became possible to calculate the starting amount of the DNA template based on the exponential phase of the PCR curve. Additionally, using the exponential phase, rather than the plateau phase, increased the dynamic range. Currently, real-time PCR analysis is usually based on the PCR crossing point (*quantification cycle*), *C*q. *C*q is defined as the PCR cycle-number at which the signal monitoring the process reaches a predefined threshold level. Older terms that have also been used as the basis for calculating the amount of DNA/cDNA starting material include *threshold cycle*, *C*_t_, and *crossing point*, *C*p (see [40,41,42]).

The sensitivity of the quantitative (q) PCR assay is dependent on the specific labeling of the DNA/cDNA. Previously, EtBr was the preferred dye because of its strong shift in fluorescent intensity when bound to DNA. However, the use of novel dyes were already beginning to make their way by the mid-1990s. One of these dyes was SYBR green I, which greatly improved sensitivity [43,44,45]. Whereas 7000 ng of 40-basepair DNA is needed to give a visible signal on a gel using EtBr, less than 14 ng is needed when using SYBR Green I. In addition, Karlsen *et al.* [43] showed that SYBR green I was less dependent on the length of the DNA, thus generating similar fluorescence levels among short and long DNA fragments. In addition to novel DNA-specific dyes, several target-specific labeling strategies have been developed for qPCR (explained in the following sections), including gene specific probes [46]. Extensive work has also been conducted to standardize qPCR procedures, including laboratory practices and data analysis (See description of the MIQE-guidelines [42]. Such advances have led to qPCR becoming the gold standard for quantifying gene expression levels, both in research and in diagnostics. 

In the present review, our main focus will be on the different strategies used for obtaining single cells or cell content from tissue slices or from dispersed cell cultures as a basis for gene expression analyses. We will then discuss strategies for optimizing RT and qPCR based on material from single cells.

While this review focuses on single-cell qPCR, several of the discussed methods are highly relevant for researchers exploring single-cell RNA-sequencing. However, we will not discuss RNA-sequencing *per se* but encourage the readers to study recent research papers and reviews specifically on this topic [47,48,49,50,51,52,53,54,55].

## 3. Single-Cell Isolation and Harvesting Strategies

Harvesting and securing the small amount of RNA molecules found within a single cell requires meticulous laboratory practice. In our laboratory we utilize separate rooms for RNA and cDNA/DNA handling. All equipment and experimental hardware are treated with RNase-inactivating reagents, like RNaseZAP (Ambion, TX, USA). In addition, we only use certified RNase-free aerosol-resistant filter tips, tubes, and reagents. All glassware is baked overnight at 220 °C, including glass capillaries used for making cell harvesting pipettes and patch electrodes. Over time, DNA contaminations may also lead to false positives. Therefore, decontamination strategies should include DNA degrading detergents.

As mentioned above, two of the initial strategies to obtain DNA or RNA from single cells used glass capillaries. These methods involve either harvesting the whole cell, or via patch clamping, harvesting only the cell’s content or cytosol [30,33]. Additional methods include isolating cells using laser-assisted micro-dissection [56,57,58,59], or by utilizing fluidics techniques, such as fluorescent-activated cell sorting (FACS) [60,61] and microfluidic technology utilizing polydimethylsiloxane (PDMS)-based lab-on-a-chip plates [62,63,64,65,66]. An overview of the different technologies are given in Table 1.

### Fluidics Technology

The user-friendly environment and high throughput of fluidics technology compared to cell and cytosol harvesting with using glass capillaries have made these methods favorable in many applications.

**Table 1:** Overview of different cell and cytosol harvesting techniques.

Method	Equipment Costs	Laboratory Skills	Throuhgput	Tissue
FACA	High	Normal	High	Dissociated cells (*in vitro*)
Microfluidics	High	Normal	High	Dissociated cells (*in vitro*)
Laser assisted microdissection	High	High	Low	Intact fixed and live tissue (*in vitro/ex vivo*)
Whole cell harvesting	Low	Normal	Medium	Dissociated cells (*in vitro*)
Harvesting of cytosol using patch pipette	High	High	Low	Intact live tissue (*in vitro/ex vivo*)

**Figure 1:**
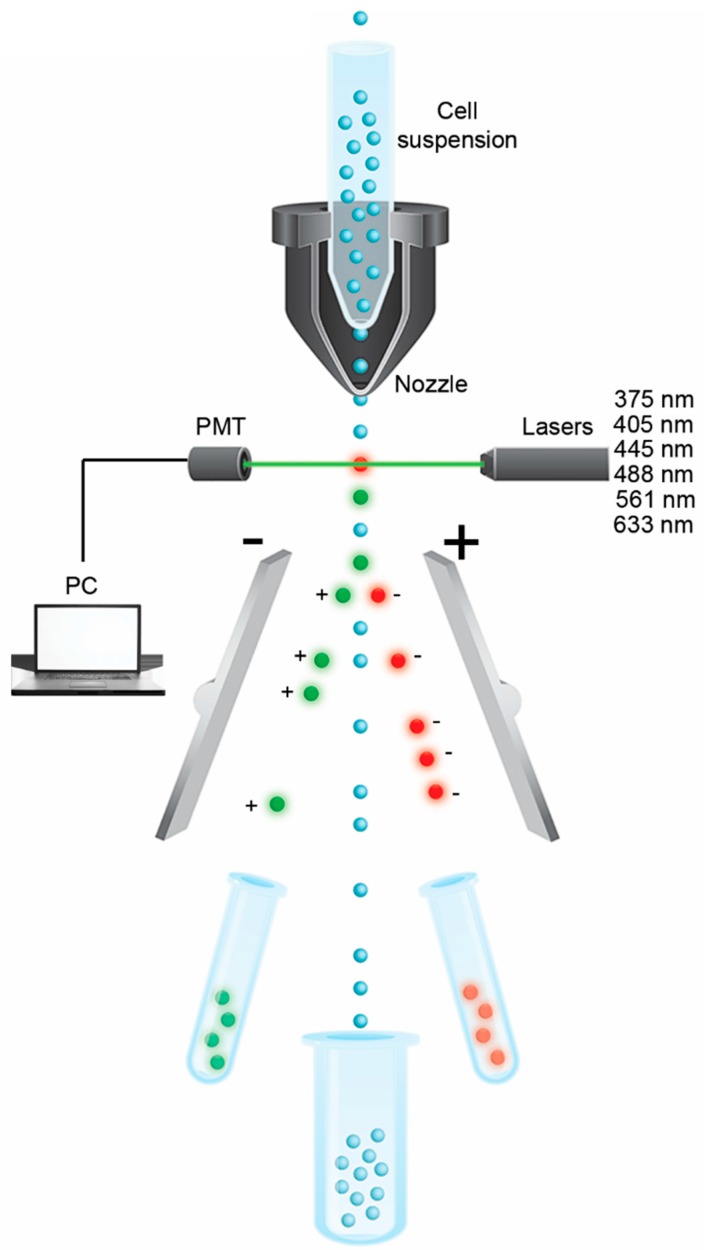
Fluorescence-activated cell sorting (FACS). The suspended cells are subjected to different fluorescent tags depending on the experimental set-up. As the cells flow in a stream of liquid they pass through a laser-detector system that monitors the fluorescent and light scatter characteristics. Based on their characteristics cells are separated in an electric field and into different collecting tubes or multiwell plates. Depending on the experiment a variety of markers may be used for separating the cells of interest. Demonstrated in this figure are cells tagged with green and red fluorescent proteins. Cells that are not labeled are separated into a third column/tube.

FACS sorting of cells allows separation or sorting of heterogeneous cells into different containers or distribution of individual cells onto multi-well plates (Figure 1). Before separation, the cells are labeled with different fluorescent probes depending on the cytometry equipment and on the experimental setup. For instance, if the setup has three lasers, up to twelve different parameters can be quantitatively assaye, including viability, apoptosis, necrosis, intracellular Ca^2+^ signaling, membrane potential, and cell cycle stage (see review by Herzenberg *et al.* [61]). The most common lasers are the 488 nm (>20 mW) and 633 nm (>18 mW). However, depending on the experiments several additional lasers may be used including 375 nm (>7 mW), 405 nm (>50 mW), and 561 nm (>18 mW). The fast flowing liquid allows for single-cell separating before passing between one or several lasers and a detector. As individual cells pass, the detector measures light scatter from the emitting fluorophores. Depending on the selected characteristics, each droplet of liquid containing a single cell is given a charge, allowing cells to be separated into separate collecting tubes by an electric field just downstream of the laser-detector system. One disadvantage of this approach is that cells or cell cultures must be subjected to stimulation experiments and treated in a separate environment before FACS analysis.

**Figure 2:**
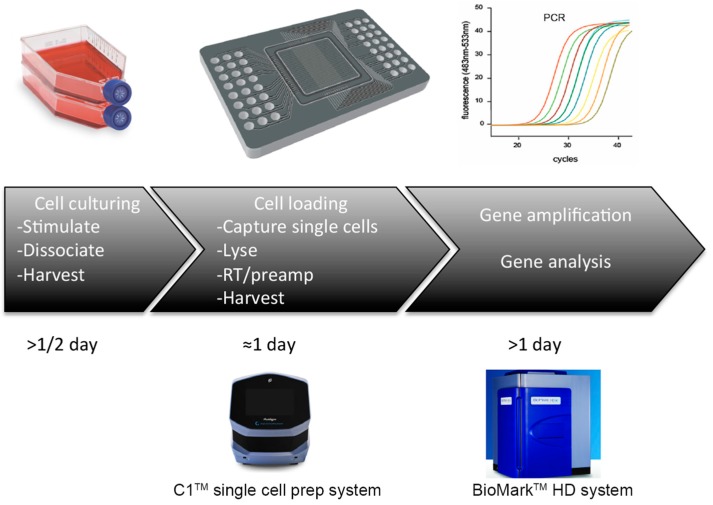
Microfluidics. Lab-on-a-chip technology allows for high-throughput screening in a microenvironment using small volume chambers to conduct different experiments. (**Upper**) Similar to FACS, the cells need to be dissociated prior to the experiments; (**Middle**) the cells are typically placed in a chamber and, depending on the technology, the cells may be separated into different chambers containing only one cell; (**Lower**) the technology currently used by Fluidigm are single-phase microfluidic systems using multilayer soft lithography to make on and off valves to compartmentalize the cells. Another promising technology is the droplet-based microfluidics to handle small volumes of fluids. (**Right**) following single-cell isolation, lysis and cDNA synthesis the samples are subjected for gene analysis. A typical qPCR profile is shown with different colors representing the amplification curve of target gene(s).

To overcome the one experiment-one machine paradigm, a novel concept of a “total chemical analysis system” (TAS) utilizing microfluidics (often termed μTAS) has emerged. The commercially available platform provided by Fluidigm is based on single-phase microfluidic systems using multilayer soft lithography (Figure 2). Multilayer soft lithography allows for compartmentalization of the cells by making and controlling small channel valves [67]. Another promising technology to handle small volumes of fluids is droplet-based microfluidics [68,69]. Depending on the technology, μTAS has the potential to provide different microenvironments where cells are grown and stimulated in small chambers whereupon either programmable valves regulate solution flow in or out of the chamber, or using droplet-based technology, the cells are guided to successive chambers for downstream experiment and analysis. These techniques may soon allow automated patch-clamp recordings and intracellular Ca^2+^ measurements [62,70], followed by transcriptome analysis in one chamber and proteomics analysis in another [66,71,72]. Microfluidics has also been applied to cells grown in monolayers or in three-dimensional environments, opening novel possibilities to explore intercellular communication.

Despite the great potential in single-cell analysis using μTAS microfluidics, the method is limited by the range of cells that can be used; in particular, the fixed chamber size found on the micro plates can limit the use of variable cell sizes. In addition, because the μTAS technology is still in its infancy, commercial systems offering multi-experimental microfluidic chips are limited to proof-of-concept. A few companies like Fluidigm have made several automated instruments for single-cell gene expression analysis, including sequencing. Their platforms are constructed of devices able to isolate single cells followed by lysis chips that can analyze gene sequence and expression from single cells.

## 4. Single-Cell Laser-Assisted Microdissection

**Figure 3:**
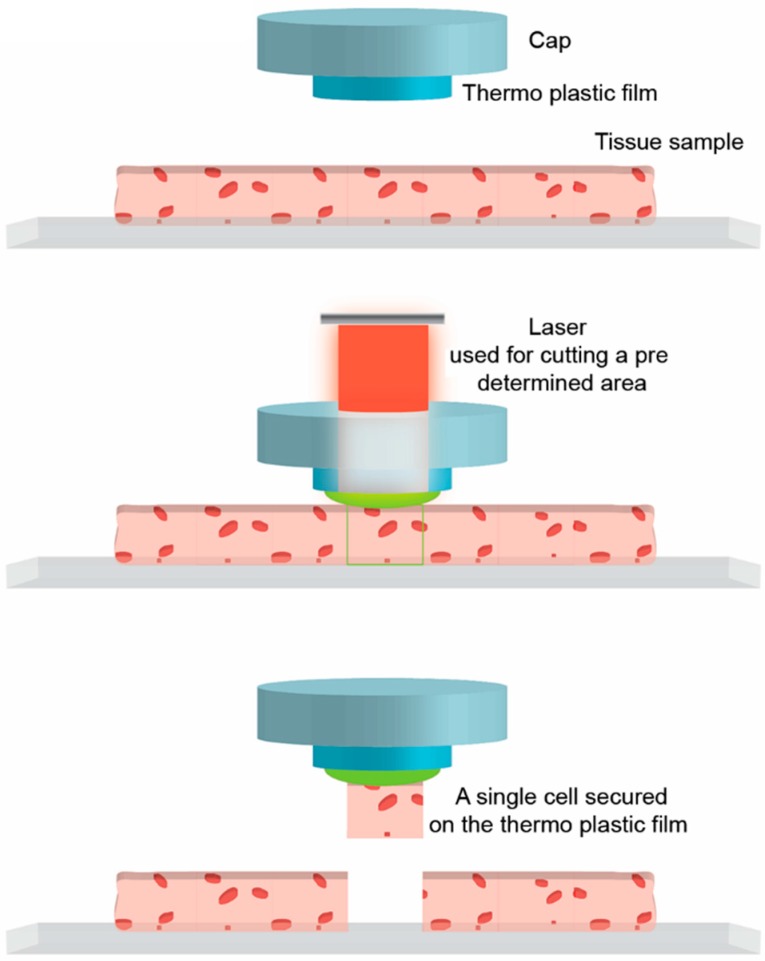
Single-cell laser-assisted microdissection. (**Upper**) The technique utilizes a laser placed on a microscope for visual identification; (**Middle** and **lower**) the laser can be controlled to dissect out target cells in fixed and live tissue. Several different technologies exist on how the laser works and how the target cell is collected following dissection.

While microfluidics are dependent on dissociated cells or cells removed from their natural environment, laser-assisted microdissection methods and cytosol harvesting through a patch-clamp pipette (see below) can be used in intact tissue [56]. In addition, laser-assisted microdissection does not require enzymatic dissociation of cells, making it less prone to disrupting intracellular signaling pathways. The cells are harvested under direct microscopic visualization and the method can be used on both live and fixed tissues (Figure 3) [57,58,59,73]. Today, there are four slightly different technologies that allow for precise laser dissection: laser microbeam microdissection (LMM), laser pressure catapulting (LPC), microdissection of membrane-mounted tissue (MOMeNT), and laser capture microdissection (LCM) (reviewed by [73]). All four technologies use a controllable pulsating laser coupled to a microscope allowing precise dissection of target cell(s). Depending on the equipment, the laser can be controlled by moving the objectives, by moving the microscope stage, or by using a dichroic mirror. The laser creates a cutting width of around 1 μm. One of the main challenges in single-cell laser assisted microdissection analysis is, therefore, the ability to dissect only the cell of interest and avoid contamination with neighboring cells or other unspecific fragments. This challenge can result in both false positives and false negatives. Dissecting too conservatively may lead to a cut not encompassing the entire cell, resulting in insufficient RNA harvest for downstream analysis. Dissecting too liberally may, on the other hand, lead to inclusion of unwanted RNA into the sample.

## 5. Harvesting Cells or Cytosol through Glass Capillary

**Figure 4:**
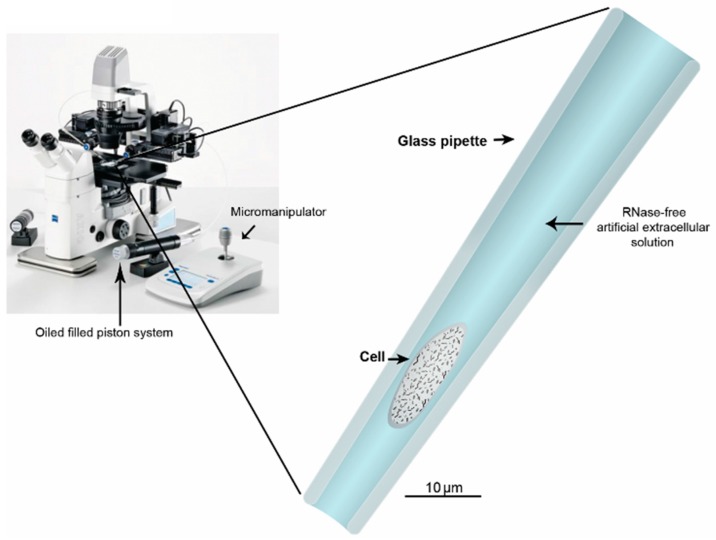
Harvesting of single cells in culture. The technique provides an economical and simple to use platform for harvesting single dissociated cells in culture. (**Left**) The cells are monitored under a microscope; (**Right**) using a glass pipette connected to a micromanipulator single cells can be collected relatively easily and transferred to a new tube for lysis and cDNA synthesis. The photograph of a typical microscope set-up is modified from Eppendorf.

Similarly to laser assisted microdissection, harvesting of whole cells or cell cytosol using a glass capillary allows direct visualization of the target cell through a microscope. The harvesting of whole cells assures complete cell isolation and minimizes loss of already-limited amounts of RNA before the RT step (Figure 4) [6,74,75,76]. The method typically uses a controllable piston system coupled to a micromanipulator [6,75]. To avoid delays when adjusting the harvesting pressure the piston system should contain a non-compressible liquid, such as mineral oil. The glass capillary used for the harvesting can be made using a horizontal or vertical puller and the final diameter should be about 1/3 of the cell diameter. The glass can also be polished using heat to avoid sharp edges. In addition, the tip can be modeled to a specific angle to improve harvesting and reduce the possibility of collecting surrounding solution. However, even with an optimized harvesting pipette, precautions should be taken. When collecting whole cells a small amount of the surrounding extracellular solution will follow into the collecting pipette [75]. This collected solution may contain contaminants like unwanted RNA. In fact, during our own work of optimizing single-cell qPCR assays we compared harvesting strategies on primary dispersed cells from pituitary with an immortalized pituitary cell line. We discovered that unwanted RNA contamination was dependent on the type of cell culture used. When harvesting whole cells from dissociated primary cell cultures there was a substantial amount of RNA present in the cellular bath. This contamination introduced false positive results in almost all of our samples. Even performing control experiments by solely resting the pipette in the bath for a few minutes had the potential of transferring unwanted RNA to our samples. However, when using the rat pituitary tumor GH4 cell line, we were able to collect whole cells without introducing false positives. We believe that these contradicting observations are a result of the relatively rough mechanical handling following chemical (trypsin, collagenase, *etc.*) treatment needed to dissociate tissues into single cells, as compared to the gentle pipetting sufficient for detaching cells like GH4 from the dish surface. In addition, most cell lines are usually well attached to plastic and glass surfaces making it easier to properly flush or wash the culture plates with clean incubation solution prior to collecting the cell of interest. Still, several groups have used whole cell harvesting on primary cell cultures seemingly without RNA contamination [6].

**Figure 5:**
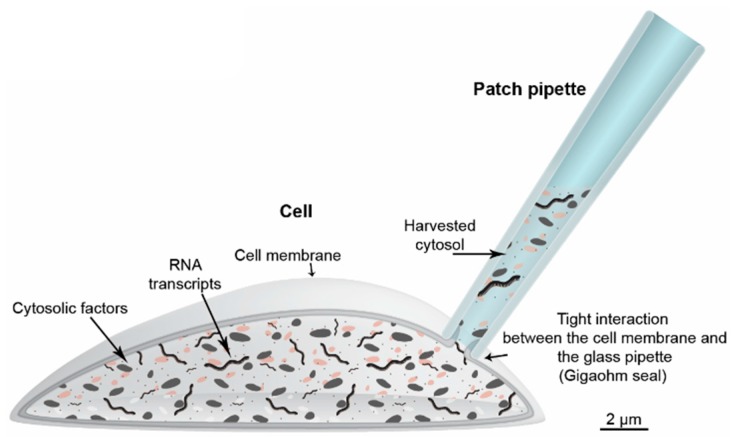
Harvesting of the cell’s cytosol using a patch pipette. The technique is usually performed on tissue slices and combines patch-clamp electrophysiological recordings with analysis of gene transcripts. Following the electrophysiological experiments the cytosol from the cell may be harvest into the pipette using gentle suction. When the harvesting is finalized the tip of the pipette is withdrawn from the cell. During the final process membrane fragments reseals the tip of the patch pipette and protects the harvested RNA from contaminations in the surrounding solution. Silanizing the glass surface will also prevent extracellular RNA from attaching to the pipette.

**Figure 6:**
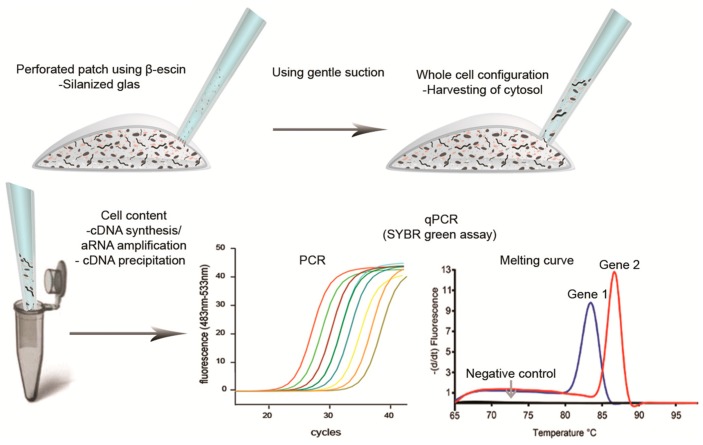
Schematic overview of the protocol used in our laboratory when performing perforated patch-clamp experiments followed by single-cell qPCR. This procedure allows for using perforated patch-clamp recordings and minimizes the incidents of false positive results during gene analysis.The patch pipette is silanized by briefly exposing the tip of the glass in a 1/10 dilution of Sigmacote. To allow for a tight high-resistance interaction between the glass and cell membrane, called a gigaohm seal, the glass tip needs to be fire polished using a microforge. (**Upper left**) The pipette is filled with an RNase-free solution suitable for the experiments and using β-escin to perforate the cell membrane; (**Upper right**) the cell cytosol can be harvested following transition to whole cell configuration using gentle suction; (**Lower left**) the cell content is transferred to a 0.5 mL tube containing RNA stabilizing solution; (**Lower middle** figure) the target genes are amplified using qPCR (colored curve represents target gene been amplified); (**Lower right**) following qPCR amplification a melting curve analysis is performed by gently heating the PCR product(s) from 65 to 98 °C while continually reading of the fluorescent (The curve(s) are plotted as the negative 1. derivative of fluorescent with respect to temperature).

To avoid aspiration of cell incubation medium or extracellular fluids, the cytosol can be harvested using the patch-clamp technique (Figure 5). Similar to harvesting of the whole cell, patch-clamping utilizes a glass pipette that is heat-pulled from a capillary to narrow the tip diameter. However, the tip is narrower than that in pipettes used for collecting whole cells. This narrow tip is positioned at the cell membrane. During optimal conditions, a tight interaction in the gigaohm range between the cell and the tip of the glass allows even small currents across the membrane to be recorded while also creating a barrier between the fluids surrounding the cell and the cell cytosol. To access the cytosol, a sub-atmospheric pressure can be created through the pipette, rupturing the membrane inside the patch. As mentioned, we experienced that RNA can attach to the glass surface and introduce false positives in the subsequent PCR analyses. To avoid this problem we silanize the patch pipette glass using Sigmacote [75]. However, combining patch-clamp experiments and subsequent single-cell RNA harvesting faces another problem. As a result of the large pipette volume, cytosolic factors are quickly diluted when using whole cell configurations. Since these cytosolic factors are important regulators of ion channel activity, researchers are often turning to the so-called perforated patch configuration where a perforating agent is added to the patch pipette. In this situation, the perforating agent makes small pores in the membrane within the patched membrane. This method leaves the cell interior preserved but does not provide access to the RNA. To overcome this obstacle and combine perforated patch-clamp recordings with subsequent cellular RNA collection, we found that substituting Amphotericin B with the saponin β-escin as the perforating agent, we could preserve the high resistance gigaohm seal when going from perforated patch to whole cell configuration. This transition was conducted in a similar way as when creating a normal whole cell configuration following formation of the gigaohm seal, simply by using gentle suction through the pipette. Thus, by combining whole cell configuration using silanized patch pipettes we were able to harvest the cell cytosol without the risk of collecting extracellular contaminants (Figure 6). Even though most of the RNA will be contained in the pipette using this approach, sample-to-sample variation can occur as a result of the harvesting. Therefore, quantitative measurements should be used with caution.

## 6. Lysis and Securing the RNA

Unless the cytosol is harvested using a patch pipette, cells need to be lysed in order to access the RNA for RT. Cell lysis must be efficient, yet not interfere with downstream processes. Today, several methods have been used for lysing single cells, The methods including optical, acoustic, electrical, mechanical, and chemical lysis (for review see [77]). The benefit of non-chemical lysis is that the methods are buffer independent. This means that the buffer can be optimized for the downstream processes such as RT. However, with the exception of chemical lysis, most of these methods are developed and validated for use with microfluidics technologies, including capillary electrophoresis. Cells collected using a glass pipette, FACS, or laser-assisted cell harvesting are typically chemically lysed, (e.g., [6,75,78,79]) where a detergent generates small pores in the membrane. Since the different detergents differ both in general structure (e.g., ionic, non-ionic and zwitterionic moieties) and their ability to interact and lyse the cell, it is important to validate and test the detergent in use. Several detergents, such as the anionic sodium dodecyl sulphate (SDS), or the cationic ethyl trimethyl ammonium bromide, lyse cells quickly, often within seconds, but also have the tendency to denature proteins potentially disturbing the RT enzyme. Other detergents are non-denaturing, including 3-[(3-cholamidopropyl)dimethylammonio]-1-propanesulfonate (CHAPS/CHAPSO), Triton, and Nonidet P-40/IGEPAL CA-630. In addition, several manufacturers deliver a variety of ready-to-use lysis buffers optimized for small number of cells, down to single-cells, in combination with RT. Several cell lysis strategies have also used high concentrations (>4 M) of guanidine salts because of their ability to inactivate nucleases and free the nucleic acids from bound proteins [80,81,82]. One disadvantage of this approach is that the RNA then needs to be purified, as a result of the detrimental effect guanidine salts have on proteins including the RT enzyme. However, Bengtsson *et al.* [83] demonstrated that low volumes and concentrations (1–2 μL and 0.5–1 M) of guanidine thiocyanate (GuSCN) efficiently lysed single pancreatic cells. Prior to the RT step, the GuSCN was diluted down to about 40 mM, thereby avoiding the need for RNA cleanup. At this concentration GuSCN even improved the conditions for RT, and the authors concluded that GuSCN serves both as a cell lysis agent and RNase inhibitor. Recently, Svec *et al.* [78] performed a comprehensive study by comparing several detergents, lysis solutions and column-based RNA isolation. The experiments were conducted using single FACS-sorted astrocytes collected into 96-well plates with 5 μL lysis buffer per well. The evaluated solutions were 7-deaza-2′-deoxyguanosine-5′-triphosphate lithium salt (100 μM), Betaine solution (4 M), bovine serum albumin (BSA) (1–4 mg/mL), guanidine thiocyanate solution (40–80 mM), GenElute linear polyacrylamide (50 ng/μL), Igepal CA-630 (0.5%–4%), and polyinosinic acid potassium salt (50 ng/μL). Interestingly, BSA was sufficient for single-cell lysis and compatible with both RT and qPCR. Earlier studies have also demonstrated that BSA efficiently buffers inhibitory factors and can improve PCR efficiency [84,85,86,87,88,89]. Since GuSCN and BSA have several positive effects downstream of cell lysis they could potentially improve conditions for single-cell analysis where only the cytosol is harvested and transferred for RT. In our experience, the cell content harvested following patch-clamp recordings may be expelled directly into a storage solution containing the relatively weak chelating agent citrate and a thermostabile RNase inhibitor. The low pH and chelating properties of citrate reduces RNA base hydrolysis. In addition, because we use random hexamers to prime the RNA for cDNA synthesis, the RNA need to be heated for several minutes at 65 °C. By using a relatively heat-stable RNase inhibitor we can add the inhibitor at an earlier step than is recommended in the protocol developed by ThermoFisher Scientific/Invitrogen. Importantly, EGTA and EDTA should be avoided because their strong chelating properties reduce free Mg^2+^ levels to below the requirements of downstream enzymes, like reverse transcriptase.

## 7. Reverse Transcription

Three basic strategies are used when priming RNA for RT are oligo(dT), random hexamer primers (or a combination of these), and gene specific primers. Earlier reports have suggested that random hexamers may be less efficient compared to oligo(dT) nucleotides that are specific for the polyA tail of mRNAs [90]. However, priming with oligo(dT) will only generate cDNA from RNA containing a polyA tail. If the starting material contains small numbers of transcripts from individual cells, and heat treatment is used for cell lysis, then the prevalence of fragmented mRNA may decrease. Random hexamers, on the other hand, will bind to all complementary regions of an RNA fragment increasing the likelihood of converting all RNA fragments into cDNA, including those targeted by gene specific primers in the subsequent PCR. A combination of the different primers may also be used and could be beneficial when performing gene analysis on single cells [91].

The amount of mRNA from single cells is limited to between 10^5^–10^6^ molecules [92] and the isolation is often time consuming, rendering the RNA from each cell valuable. Therefore, to avoid multiple sampling in order to analyze several genes from a single cell type, pre-amplification is often necessary. As mentioned above, two strategies are commonly used for increasing the number of transcripts. In the strategy developed by Vangelder *et al.* [32], the authors used oligo(dT) primers comprising a promoter recognized by the bacteriophage DNA-dependent RNA polymerase T7. Following cDNA synthesis RNase H hydrolyzes the template RNA leaving single stranded cDNA. Under isothermal conditions, the T7 synthesizes a new RNA strand from the cDNA template. Since only the initial cDNA template contains the T7 promoter, the template concentration itself does not increase, making the amplification process essentially linear. New and improved promoters and reaction buffers for RNA amplification have reduced nonspecific activity and increased cDNA yield (see e.g., [93,94,95,96,97]). However, pre-amplification involves multiple steps and is, therefore, labor intensive. A less time consuming strategy is to use two rounds of PCR, as demonstrated by Lambolez *et al.* [34]. However, in their study they could only target a few genes defined by the primers [34]. Therefore, they developed an improvement of the method using homomeric tailing of the cDNA with polyA and subsequent PCR (global amplification) with oligo(dT) primers [31,98]. In fact, Iscove *et al.* [99] demonstrated that by using this strategy they could preserve abundance relationships through amplification as high as 3 × 10^11^-fold. Further, compared to linear amplification strategies, the RNA needed for microarray analysis could be reduced by a million-fold and give reproducible results using the picogram range of total RNA obtainable from single cells. Several reports have tested and validated different amplification strategies [100,101] including the so-called switching mechanism of 5′end of RNA template PCR (SMART PCR) [102]. Both linear pre-amplification and SMART PCR have been used in single-cell RNA sequencing experiments [103,104,105,106,107].

## 8. qPCR

Several detection formats can be used in qPCR. These include fluorescent dyes, such as SYBR green, which bind to any double-stranded DNA, [90], and sequence-specific probes (see review by [108]). The advantages of using probes are that fluorescence is emitted only during specific binding and that several genes can be detected in the same reaction [109]. The main disadvantages are the cost and the fact that a melting curve analysis (explained below) cannot be performed directly following PCR. SYBR green or other non-specific DNA-binding fluorescent dyes, on the other hand, may be used with any gene-specific primer pair. Compared to probe-based qPCR, the widely-used SYBR green is more cost-efficient. SYBR green binds to the minor groove in double-stranded DNA and, once bound, the signal increases 1000 times compared to free dye in solution. As SYBR green binds to any double-stranded DNA, including primer dimers, the qPCR assay must be carefully validated. To discriminate different products, a melting curve analysis of the products is usually performed directly after the PCR without breaking the sealed samples, eliminating carry-over contamination or pipetting errors. The melting temperature of the PCR product is based not only on the product size, but also on the GC content and the distribution of GC within the PCR product. This is favorable compared to gel-electrophoresis, which can only separate the products based on size. The specificity of melting curve analysis reduces the risk of false positives and can be used to separate products with minor differences, such as point mutations.

Specificity, sensitivity, and efficiency of qPCR are dependent on numerous factors including priming strategies, purity of cDNA, as well as number and length of the PCR cycle(s). In our laboratory we utilize the freeware Primer3plus [110] to design gene-specific primers. In addition, we routinely perform *in silico* testing of all primers using software like Vector NTI [111] or similar Following initial screening, the primers are validated and the optimal primer annealing temperature is determined using cDNA synthesized from total RNA extracted from tissue. In general, lowering the annealing temperature increases sensitivity and efficiency. However, too low of a temperature can create nonspecific primer binding and give false positives. These parameters are measured using serial dilution curves of cDNA. The Cq can be plotted against the logarithm of the relative concentration of the cDNA starting material. The efficiency of the qPCR assay can then be described by the slope of the regression line (efficiency = 10^−1^/slope). If the slope of the dilution curve is −3.32, the efficiency equals 2, meaning that each PCR cycle doubles the product. If the efficiency is 2, or 100%, a 10× dilution of cDNA starting material will give a change in Cq (∆Cq) of 3.2.

Due to the limited amount of transcript, single-cell qPCR is often conducted using undiluted cDNA as template. This can result in accumulated levels of DTT and RT enzyme, which inhibit and profoundly affect the qPCR assay performance [112,113,114]. To avoid these inhibitory factors, a protocol for single-cell cDNA precipitation was developed by Liss [113]. Introducing this cDNA precipitation step into our own single-cell analyses has greatly reduced the incidence of inconclusive qPCR results. Notably, adding a known concentration of non-expressed synthetic RNA-spike can be used for validating the workflow process downstream of cell lysis including the precipitation.

## 9. Quantitative Gene Analysis

The nature of gene regulation within a single cell prevents relative quantification normalized to so-called housekeeping genes. For reliable quantification Bengtsson *et al.* [83] developed a protocol for absolute quantification based on a known standard. The genes of interest are cloned and amplified by PCR before determining the concentration spectrophotometrically (A260). A series of dilutions is made before qPCR with the diluted DNA as template. The template needs to be pure and the copy number can be determined by using the average weight of a base (660 g/mol).

## 10. Future Possibilities and Challenges

Although much progress has been made during recent years in single-cell gene analysis, the field is still facing several challenges related to harvesting strategies, and to transcript amplification and analysis. Common to most stages and technologies is the need for improved reagents and more precise enzymes, e.g., reagents that avoid or reduce the potential for biased or non-linear pre-amplification of the transcripts. In addition, more powerful software focusing on genetic analysis of single-cell transcript variability needs to be developed. 

Despite the challenges, the field of single-cell gene analysis is moving forward rapidly with continuous development of new hardware, software and reagents. In particular, we have seen a dramatic development in the field of nucleic acid sequencing. This development has resulted in more than 10-fold reduction in costs for sequencing during the last decade. In parallel with this development integrated systems, like lab-on-a-chip technology, has facilitated single-cell analysis. Within the next decade multifunctional equipment, based on microfluidics technology, will probably reduce hands on time for sample preparation and create a more streamlined processing. The working platforms will likely perform several subsequent steps including cell stimulation and manipulation, automated patch-clamp electrophysiology, imaging, including Ca^2+^ measurements, nucleic acid amplification and sequencing, and possibly proteomics, again, reducing hands-on time related to manual transfer of samples between equipment. [62,70]. 

Despite the promising and broad applications of fluidics technology, it will most probably be limited to dissociated cells in suspension. Investigations on whole organ function and plasticity require that spatial integrity of the tissue is preserved, allowing investigations of temporal events. Intact, model organs generally rule out fluidics systems. However, as opposed to the rapid development of microfluidics, the development of equipment that allows single-cell harvesting from intact tissue has relied on older techniques, like harvesting of cytosol through a patch pipette. This slow progress may now come to a close with nanotechnology. Currently, the preferred method for isolating transcripts from live, intact tissues is harvesting of the cell cytosol using a glass patch pipette. Even though this has proved valuable, it is limited by the fact that the harvesting requires a continuous tight interaction between the tip of the glass and the cell membrane, which is often lost during harvesting (Figure 5). However, recent developments demonstrate the potential use of multiwalled carbon nanotubes mounted at the tip of conventional micropipettes [115]. Because these carbon nanotubes only have a fraction of the diameter used when making conventional patch-clamp pipettes, the nanotubes can, in a less invasive fashion, access the cytosol by penetrating the cell membrane without destroying the cell. However, the technology is still at the stage of “proof of principle”, but has successfully been demonstrated to work as a cell-specific delivery system and used for electrophysiological experiments, e.g., [115,116]. With further development these tools may soon be commercially available.

## 11. Summary

Single-cell gene analysis is a highly-powerful approach to understand the dynamics of gene regulation. Depending on the research focus, several methods are available for harvesting or isolating single-cell RNA. The methods need to be carefully evaluated and considerations, like spatial and temporal gene regulation, can be affected by the chosen harvesting strategy. In addition, the sensitivity of PCR makes it prone for false positives, affecting the assay both qualitatively and quantitatively. In our experiments, in which the assay was designed for phenotyping cells based on their gene expression, we identified extracellular contamination that greatly affected the qPCR assay. Thus, thorough validation of the cell isolation process is as crucial as the validation of downstream processes.
